# TIM-Finder: A new method for identifying TIM-barrel proteins

**DOI:** 10.1186/1472-6807-9-73

**Published:** 2009-12-14

**Authors:** Jing-Na Si, Ren-Xiang Yan, Chuan Wang, Ziding Zhang, Xiao-Dong Su

**Affiliations:** 1 State Key Laboratory of Agrobiotechnology, College of Biological Sciences, China Agricultural University, Beijing 100193, China; 2 National Laboratory of Protein Engineering and Plant Genetic Engineering, College of Life Sciences, Peking University, Beijing 100871, China

## Abstract

**Background:**

The triosephosphate isomerase (TIM)-barrel fold occurs frequently in the proteomes of different organisms, and the known TIM-barrel proteins have been found to play diverse functional roles. To accelerate the exploration of the sequence-structure protein landscape in the TIM-barrel fold, a computational tool that allows sensitive detection of TIM-barrel proteins is required.

**Results:**

To develop a new TIM-barrel protein identification method in this work, we consider three descriptors: a sequence-alignment-based descriptor using PSI-BLAST e-values and bit scores, a descriptor based on secondary structure element alignment (SSEA), and a descriptor based on the occurrence of PROSITE functional motifs. With the assistance of Support Vector Machine (SVM), the three descriptors were combined to obtain a new method with improved performance, which we call TIM-Finder. When tested on the whole proteome of *Bacillus subtilis*, TIM-Finder is able to detect 194 TIM-barrel proteins at a 99% confidence level, outperforming the PSI-BLAST search as well as one existing fold recognition method.

**Conclusions:**

TIM-Finder can serve as a competitive tool for proteome-wide TIM-barrel protein identification. The TIM-Finder web server is freely accessible at http://202.112.170.199/TIM-Finder/.

## Background

Proteins have complex three-dimensional (3D) shapes, a fact well demonstrated by more than 60,000 experimentally determined structures deposited in the current PDB database http://www.rcsb.org/pdb/home/home.do. The number of unique protein folds (or architectural types) should be much smaller than the number of protein families defined by sequence similarity [1]. As more structures are determined, it also becomes increasingly clear that the distribution of proteins between different folds is not even [2]. Although many folds have so far been observed for only a few proteins, some protein folds (known as superfolds) occur frequently. As reported by Salem et al. (1999), the top ten superfolds could account for approximately one third of all proteins in the PDB database.

**Figure 1:**
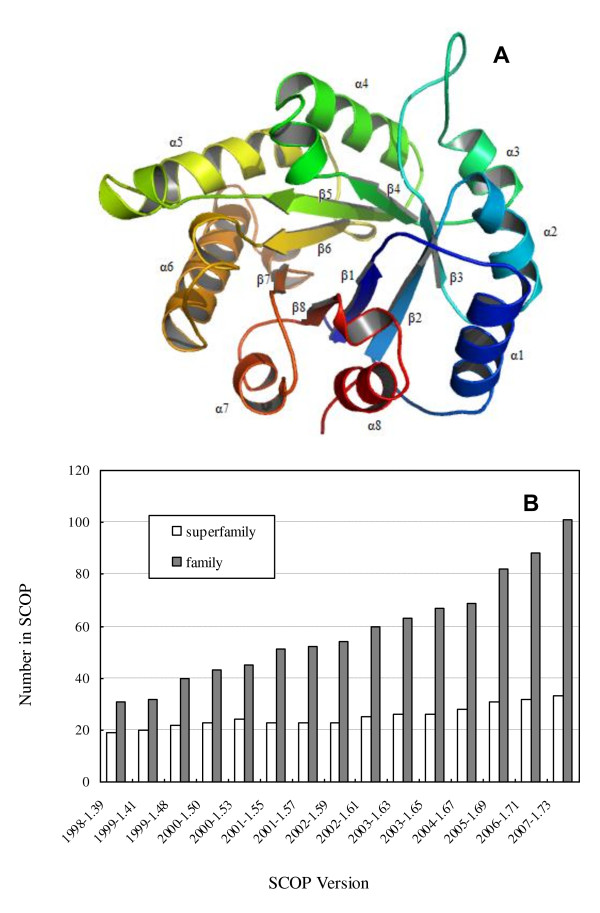
**The TIM-barrel fold**. (a) Cartoon representation of the 3D structure of a typical TIM-barrel protein (triosephosphate isomerase, PDB entry: 8tim). (b) The SCOP statistics on the TIM-barrel fold.

One of the top ten superfolds is the triosephosphate isomerase (TIM)-barrel fold (Figure 1A). It was first observed in triosephosphate isomerase and consists of eight α-helices on the outside and eight parallel β-strands on the inside that alternate along the peptide backbone [3]. In the past, many protein structures with the TIM-barrel fold have been determined, which allow a more complete understanding of the fold space of the TIM-barrel (Figure 1B). In the SCOP database (version 1.73) [4], the TIM-barrel fold contains 33 superfamilies and 101 families (Figure 1B). As a common fold with multiple functions, TIM-barrel proteins often function as enzymes. They can catalyze five of the six categories of biochemical reactions [5]. The evolution of the TIM-barrel fold has also received considerable attention, and it has been established that the TIM-barrel fold is one of the most ancestral folds [6].

To identify the structural fold for a query protein sequence, classical sequence similarity searching methods (e.g., BLAST [7] and FASTA [8]) can be employed to scan the query protein sequence against others with known structures. It is possible, however, that two structurally similar proteins may share weak sequence similarity (i.e., remote homology). Marked improvements in detecting such remote homology relationships can be obtained using sensitive sequence-searching methods such as PSI-BLAST [9] and Hidden Markov Models(HMM) [10]. In recent years, more powerful remote homology identification techniques called fold recognition or threading methods (e.g., FFAS03 [11], 3D-PSSM [12], Fugue [13], mGenThreader [14], ORFeus [15]) have been elegantly developed as well. The overall impressive performances of these algorithms, which combine different types of structural and sequence information, have been widely demonstrated in a series of CASP experiments [16], as well as in some real-time evaluation systems of structure prediction servers (e.g., LiveBench) [17].

The advantage of the above methods is that they are suitable for many protein fold types, but they may lack the specificity to recognize certain folds. Therefore, it is necessary to develop specialized computational tools for recognizing some important protein folds. Similar efforts have been successful in identifying some protein families, such as β-barrel membrane proteins [18-21], G-protein coupled receptors (GPCRs)[22,23] and glycosyltransferases [24]. To accelerate the exploration of the sequence-structure protein landscape in the TIM-barrel fold, it is necessary to develop a specific and reliable method to detect TIM-barrel proteins.

In this work, any measurement between two proteins can be regarded as a descriptor. For instance, the *e-*value obtained from a BLAST search of protein *A *against protein *B *can be regarded as a descriptor between them. Based on such a broad definition, a great many descriptors have been developed in past decades, of which many can be used to measure the sequence similarity between two proteins. Because different descriptors may reflect different aspects of similarity between two proteins and can be complementary to a certain extent, the combination of well-performing descriptors can result in improved performance. An example of such improvement is the generic fold recognition method developed in our previous work [25]. Based on a similar strategy, in this work we combined three descriptors into a prediction system with the assistance of Support Vector Machine (SVM). The three implemented descriptors are the sequence-alignment-based descriptor using PSI-BLAST *e*-values and bit scores, the descriptor based on the alignment of secondary structural elements (SSEA), and the descriptor based on the occurrence of PROSITE functional motifs [26]. The proposed TIM-barrel protein identification system, TIM-Finder, gives highly accurate results. The details of the construction of the three descriptors and the SVM-based predictor are reported. The overall performance of TIM-Finder is also benchmarked against one of the state-of-the-art fold recognition methods, Fugue, via a proteome-wide identification of TIM-barrel proteins in the bacteria *Bacillus subtilis*.

## Results and discussion

### Performance of the individual descriptors

In the present study, three descriptors were used to recognize TIM-barrel proteins. The three descriptors were individually benchmarked via a reference dataset called SCOP_10_mod, which contains 163 TIM-barrel proteins and 843 structurally diverse non-TIM-barrel proteins. The details of the construction of the three descriptors, the compilation of the SCOP_10_mod dataset, and the evaluation procedures are outlined under Methods.

**Figure 2:**
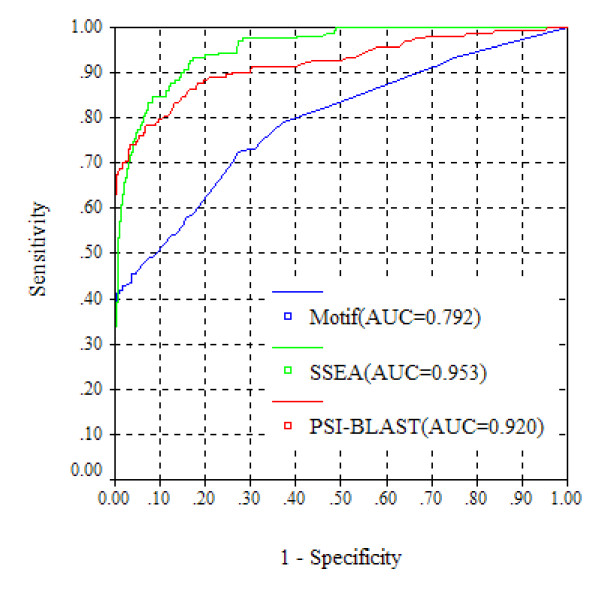
**The overall performance of three descriptors individually measured by ROC analysis**.

The overall performance of the PSI-BLAST-based descriptor was measured using Receiver Operator Characteristic (ROC) analysis [27], which plots true positive rate (TPR) (i.e., Sensitivity) as a function of false positive rate (FPR) (i.e., 1-Specificity). The area under the ROC curve (AUC) was also employed to assess the performance. As shown in Figure 2, the PSI-BLAST-based descriptor results in an AUC value of 0.920. At a 5% FPR control, the PSI-BLAST-based descriptor can correctly detect 74.8% of TIM-barrel proteins. As a profile-based sequence searching algorithm, PSI-BLAST has been widely applied in many aspects of protein structure and function prediction. For instance, the PSI-BLAST algorithm has been integrated into most state-of-the-art fold recognition methods [12-14]. It also acts as a reference algorithm to benchmark any newly developed fold recognition method. In this work, the PSI-BLAST-based descriptor was used as a key component to construct our TIM-barrel protein prediction system.

**Figure 3:**
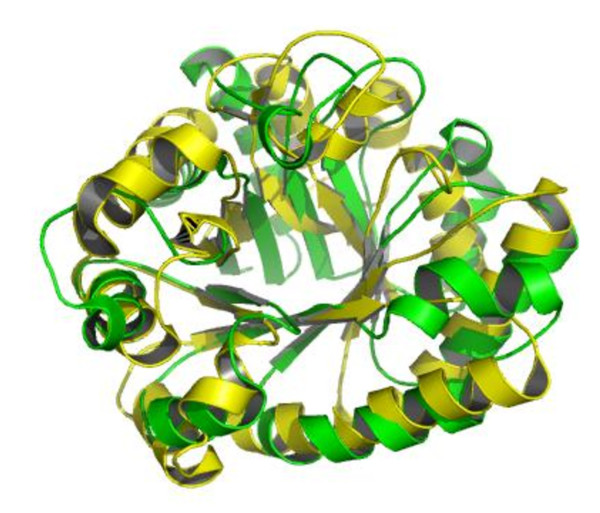
**The CE structural alignment of two TIM-barrel proteins **1vpqA** and **1i60A. Although the PSI-BLAST-based descriptor was not able to detect the remote homologous relationship between 1vpqA (green) and 1i60A (yellow), the SSEA-based descriptor can successfully recognize their structural similarity based on a SSEA score of 0.814.

Predicted secondary structure has long been proven to be helpful in protein fold classification and recognition [28], and the SSEA-based descriptor has been reported to be an effective way to consider the information of predicted secondary structure [14,29,30]. As shown in Figure 2, the SSEA-based descriptor performs the best, and it achieves an AUC value of 0.953. At a FPR less than 5%, the SSEA-based descriptor is able to successfully recognize 78.5% of the TIM-barrel proteins. As reported in our previous study [25], the PSI-BLAST-based descriptor is much better than SSEA at generic fold recognition. Interestingly, SSEA is more powerful than the PSI-BLAST-based descriptor in recognizing TIM-barrel proteins. Generally, the TIM-barrel fold has a well conserved 3D structure, which consists of eight β-strands and eight α-helices. From N-terminus to C-terminus, the secondary structure of a typical TIM-barrel fold is strictly arranged as β1-α1-β2-α2-β3-α3-β4-α4-β5-α5-β6-α6-β7-α7-β8-α8 (Figure 1A), which may explain why the SSEA descriptor is so powerful in recognizing TIM-barrel proteins. The performance of the SSEA-based descriptor is further demonstrated in two TIM-barrel proteins distant from one another in sequence space: 1vpqA (SCOP index: c.1.32.1) and 1i60A (SCOP index: c.1.15.4). Because the two proteins share a weak sequence similarity, the PSI-BLAST-based descriptor fails to recognize their remote homologous relationship. With a SSEA score of 0.814, however, the SSEA-based descriptor is able to catch these two proteins' structural similarity. The success of SSEA should be ascribed to the overall conservation of secondary structure topology between these two proteins, which can be observed from their structural alignment derived from the CE algorithm [31] (Figure 3).

**Figure 4:**
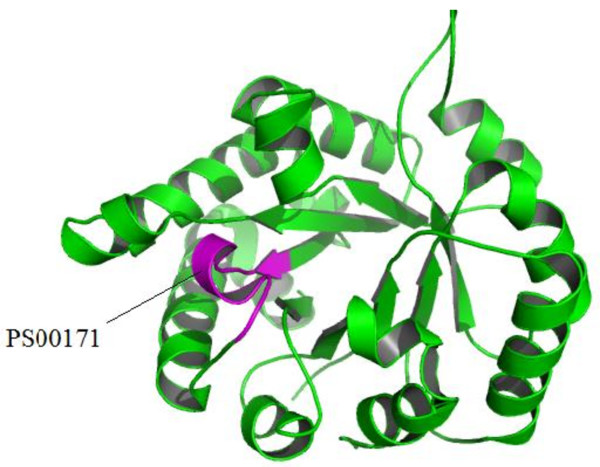
**Cartoon representation of a TIM-barrel protein (PDB entry: **1n55**)**. The structural location of the most frequently occurred PROSITE motif (entry: PS00171, pattern: [AVG]- [YLV]-E-P- [LIVMEPKST]- [WYEAS]- [SAL]- [IV]- [GN]- [TEKDVS]- [GKNAD]) in the 3D model is shown in magenta.

The motif-based descriptor leads to an AUC value of 0.792, which is less impressive than the PSI-BLAST- and SSEA-based descriptors (Figure 2). At a ≤ 5% FPR control, the motif-based descriptor only correctly recognizes 46.0% of the TIM-barrel proteins. Sequence motifs have been reported to correlate with protein folds [25,32]. The central idea of the motif-based descriptor is to recognize TIM-barrel proteins based on motif-fold compatibility. In this work, we used the PROSITE database, because it is one of the most widely used and comprehensive sequence motif databases. The PROSITE motifs are mainly defined as patterns (i.e., regular expressions) and profiles, which were derived from analysis of sequences of known function. For each PROSITE motif, its compatibility with the TIM-barrel fold was measured by a score called *S*(*TIM*|*motif*). Of the 2096 motifs under investigation, 103 have *S*(*TIM*|*motif*)> 0.1, including 91 patterns and 12 profiles. As an illustrative example, we have provided the 3D model for a TIM-barrel protein and the structural location of a PROSITE motif PS00171 (Figure 4), which was analyzed as having the highest *S*(*TIM*|*motif*) score. Due to the functional diversity of TIM-barrel proteins, the PROSITE motifs are obviously enriched in this fold. Therefore, the motif-based descriptor, which represents local sequence features of proteins, should be particularly suitable for recognizing TIM-barrel proteins. Additionally, the motif-based descriptor is alignment independent, meaning that it should be complementary to the other two alignment related descriptors (i.e., the PSI-BLAST- and SSEA- based descriptors). Thus, it should be informative when combined with the other two descriptors, although the motif-based descriptor itself is not powerful.

### Performance of TIM-Finder

**Figure 5:**
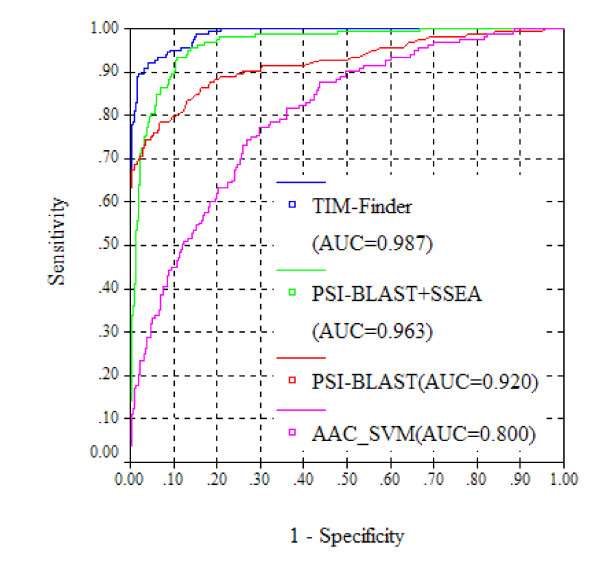
**The overall performance of TIM-Finder measured by ROC analysis**.

Using SVM, the PSI-BLAST-, SSEA- and motif-based descriptors were combined into a prediction system called TIM-Finder. More details of the construction of TIM-Finder are available under Methods. The overall performance of TIM-Finder was further measured by the ROC curve (Figure 5). For the purpose of comparison, prediction based on the combination of PSI-BLAST- and SSEA-based descriptors was also carried out. Meanwhile, the result from the single PSI-BLAST-based descriptor is also shown in Figure 5 to provide a benchmark for TIM-Finder. As shown in Figure 5, TIM-Finder results in a high AUC value of 0.987. Since the performance at low false positive rates is more important for real-world applications, the sensitivity values of TIM-Finder at 1%, 5% and 10% FPRs are further listed in Table 1. With a 5% FPR rate control, TIM-Finder is able to correctly identify 92.0% of the TIM-barrel proteins, which is approximately 17 percentage points higher than the individual PSI-BLAST-based descriptor and about 12 percentage points higher than the combination of the PSI-BLAST- and SSEA-based descriptors (Table 1; Figure 5). Although the motif-based descriptor itself has an overall weak performance, it should be emphasized here that the motif-based descriptor does make an important contribution to the final performance of TIM-Finder (Figure 5), implying that it relies on quite different features from the PSI-BLAST- and SSEA-based descriptors. Generally, TIM-Finder has been benchmarked to have an excellent performance, implying it can be applied in practical use such as proteome-wide TIM-barrel protein detection.

**Table 1:** The sensitivity values of TIM-Finder at different false positive rates (FPRs)^a^

	Sensitivity		
	
	FPR = 1%	FPR = 5%	FPR = 10%
PSI-BLAST	68.7%	74.8%	79.8%
PSI-BLAST + SSEA	39.3%	80.3%	89.6%
TIM-Finder	**80.4%**	**92.0%**	**95.1%**
AAC_SVM	12.9%	31.9%	44.8%

^a ^The FPRs at 1%, 5% and 10% mean that the corresponding specificity values are 99%, 95% and 90%, respectively.

### Comparison with the amino acid composition based SVM model

As reported in the literature [33,34], simple amino acid composition (AAC) based SVM models have been widely employed for classification of proteins. For comparison, a simple composition based method (AAC_SVM) was also developed to distinguish TIM-barrel and non-TIM-barrel proteins. More details about the construction of AAC_SVM are available in Methods. Due to the limited sequence information encoded by AAC, the performance of AAC_SVM tends to be worse than TIM-Finder (Table 1; Figure 5). AAC_SVM achieves an AUC value of 0.800, which is much lower than that of TIM-Finder (0.987) (Figure 5). At a 5% FPR control, AAC_SVM can correctly detect only 31.9% of TIM-barrel proteins, while the corresponding identification rate of TIM-Finder is up to 92.0% (Table 1).

### Comparison with the Fugue fold recognition method

As mentioned, TIM-barrel proteins can also be identified by state-of-the-art fold recognition methods. Therefore, it is also important to benchmark TIM-Finder against fold recognition methods. In this work, TIM-Finder was benchmarked against the Fugue fold recognition method, a profile-based fold-recognition program that makes extensive use of both sequence and structural information [13], via a proteome-wide TIM-barrel protein identification in *B. subtilis*. For the purpose of comparison, TIM-barrel protein identification based on a standard PSI-BLAST search was also carried out. More details about the proteome-wide computational experiments are available in Methods.

Of the 3,575 *B. subtilis *protein sequences under investigation, TIM-Finder detects 194 TIM-barrel proteins at a 1% FRP control (Table 2). At the same confidence level, Fugue and PSI-BLAST recognize 184 and 164 TIM-barrel proteins, respectively. At a 5% FPR control, the performance of TIM-Finder is still better than that of Fugue and PSI-BLAST. Although Fugue has been well benchmarked to be a leading fold recognition method [17,35], TIM-Finder revealed an overall higher TIM-barrel protein identification rate than Fugue, confirming that it is really necessary to develop specific recognition methods for some important protein folds. It is not surprising that both TIM-Finder and Fugue can surpass the PSI-BLAST search, because the PSI-BLAST search was incorporated in TIM-Finder as well as in Fugue. It was also observed that the three methods are complementary to some extent (Table 3). Considering the identification at a 1% FPR control, for instance, only approximately 80% of the TIM-barrel proteins identified by TIM-Finder are also recognized by Fugue. To maximize a proteome-wide TIM-barrel protein identification, a combination of different methods could therefore still be recommended.

**Table 2:** Proteome-wide TIM-barrel protein identification in *B. subtilis*

	Identified TIM-barrel proteins in *B. subtilis*		
	
	TIM-Finder	Fugue	PSI-BLAST
99% confidence^a^	**194/3,575 = 5.4%**	184/3,575 = 5.1%	164/3,575 = 4.6%
95% confidence^b^	**294/3,575 = 8.2%**	280/3,575 = 7.8%	250/3,575 = 7.0%

^a^A 99% confidence level means a FPR rate ≤ 1% (i.e., Specificity = 99%). ^b^A 95% confidence level means a FPR rate ≤ 5% (i.e., Specificity = 95%).

**Table 3:** Comparison of the consensus among TIM-Finder, Fugue, and PSI-BLAST in detecting TIM-barrel proteins^a, b^

	TIM-Finder	Fugue	PSI-BLAST
TIM-Finder	--	154 (79.4%)	144 (74.2%)
Fugue	154 (83.7%)	--	143 (77.7%)
PSI-BLAST	144 (87.8%)	143 (87.2%)	--

^a ^The comparison was based on a 99% confidence level.^b ^The value outside the parentheses denotes the total number of *B. subtilis *sequences that were identified as TIM-barrel proteins by both methods, whereas the value inside the parentheses represents the corresponding percentage, defined as the total number of consensual predictions from two methods divided by the total number of TIM-barrel proteins identified by the single method appearing in the row.

However, the assessment of different methods based merely on the number of identified TIM-barrel proteins in *B. subtilis *is still quite subjective. In this work, the following efforts were made to allow a fair comparison. First, the same NR database (i.e., NR90) was used in processing the above three methods. Second, all TIM-barrel proteins in the Fugue library (i.e., the HOMSTRAD database) share sufficient sequence similarity with the TIM-barrel proteins in the library of TIM-Finder (i.e., the SCOP_40_TIM dataset), ensuring a fair comparison between TIM-Finder and Fugue. Even with the above efforts, however, we are still not able to guarantee a fully unbiased assessment. For instance, the Fugue Z-score threshold for different confidence levels was proposed by considering the recognition of all protein fold types, which may not be suitable for the recognition of TIM-barrel proteins alone.

## Conclusions

The proposed method TIM-Finder, incorporating the PSI-BLAST-, SSEA-, and motif-based descriptors, has been intensively benchmarked to have good performance, suggesting that it can serve as a powerful predictor to be practically applied in proteome-wide TIM-barrel protein detection. Concerning future development, the following three aspects should be taken into account to obtain a more comprehensive prediction system. 1) From the viewpoint of structural biologists, it may be more interesting to target new TIM-barrel superfamily proteins. Therefore, in the future version of TIM-finder, we may consider including a prediction option to indicate whether a query sequence belongs to a new TIM-barrel superfamily. 2) The current TIM-Finder is not able to provide a sequence alignment between the query sequence and the generated hit, which may limit its further application. To solve this problem, a state-of-the-art profile-profile alignment algorithm [36] can be employed. 3) The current TIM-Finder may lose some sensitivity in processing sequences with multiple domains. Therefore, a reasonable domain parser should be added as a preprocessing step in the future version of TIM-Finder.

## Methods

### Data sets

In the present study, we used the SCOP database (version 1.73; released in December, 2007) to assess the performance of the different descriptors, train the SVM models of TIM-Finder, and construct the library of TIM-Finder. Several SCOP sequence datasets with different sequence redundancy were obtained from http://scop.mrc-lmb.cam.ac.uk/scop/[4,37]. The downloaded SCOP_10 dataset contains 163 TIM-barrel proteins and 5,451 non-TIM-barrel proteins, and the sequence identity for any sequence pair in this dataset is ≤ 10%. Because all TIM-barrel proteins have a sequence length of more than 100 amino acids, the non-TIM-barrel proteins with less than 100 amino acids were removed. Moreover, for each non-TIM-barrel fold, only one protein was randomly selected as the final negative control. Thus, the SCOP_10 dataset was compiled into a modified dataset of 163 TIM-barrel proteins and 843 non-TIM-barrel proteins (i.e., SCOP_10_mod), which was employed to assess the performance of the different descriptors as well as training the SVM models. The SCOP_40 dataset, containing 9,536 proteins, was downloaded for the construction of the library of TIM-Finder. The downloaded SCOP_95 dataset, containing 15,273 proteins, was used to derive the motif-based descriptor.

The NCBI non-redundant (NR) sequence database was downloaded from ftp://ftp.ncbi.nlm.nih.gov/blast/ (November, 2008). The NR database was further clustered at 90% identity by using the CD-hit program [38], and the resulting NR90 database, containing 4,205,215 sequences, was used to implement the PSI-BLAST search. To derive the motif-based descriptor, the PROSITE release 20.27, which contains 1,318 patterns and 778 profiles, was obtained from http://www.expasy.org/prosite/[26].

### Descriptors

#### PSI-BLAST-based descriptor

A PSI-BLAST search for sequence *A *against sequence *B *was executed in the following two steps. First, sequence *A *was searched against the NR90 database by PSI-BLAST for three rounds to generate a profile. The *e*-value cutoff for including sequences in the profile was set at 0.001. Second, a PSI-BLAST search was performed on the obtained profile against sequence *B *for another round. The above PSI-BLAST search resulted in two parameters, the expected value *evalue(A, B) *and the bit score *Score(A, B)*, which can be used to measure the sequence similarity between *A *and *B*. In this work, *evalue(A, B) *was modified according to the following equation.

#### Secondary structure element alignment-based descriptor

Briefly, performing a SSEA for two query sequences *A *and *B *consisted of the following three procedures. First, the secondary structure prediction for the two query sequences was carried out by PSIPRED [39]. Second, the predicted secondary structural string was converted into a secondary structure element such that "H" represents a helix element, "E" denotes a strand element, and "C" stands for a coil element. For instance, the secondary structure string HHHHHHHCCCCEEEEEEECCCCCCCHHHHHH should be shortened to HCECH, the length of each element being retained for the scoring of SSEA. Third, the two shortened strings (i.e., secondary structure elements) were aligned using a dynamic programming algorithm [40] with a scoring scheme adapted from Przytycka et al. [30]. The resulting alignment score *SSEA(A, B)*, ranging from 0 to 1, was used as the descriptor of the similarity between two query sequences. To derive the SSEA-based descriptor, our in-house SSEA algorithm was implemented. More details about this SSEA algorithm are available in our previous study [25].

#### Motif-based descriptor

In this work, the PROSITE motif library was used to derive the motif-based descriptor. First, the correlation between each PROSITE motif presence and the TIM-barrel fold in the SCOP database (i.e., SCOP_95) can be quantified by a log-odds score *S *defined as:

where *p*(*motif*) and *p(TIM) *are the individual probabilities of finding a particular sequence motif and a TIM-barrel protein in the SCOP database, and *p*(*TIM*, *motif*) is the corresponding joint probability. We used the Perl script ps_scan ftp://ftp.expasy.org/databases/prosite/tools/ps_scan/ to compute whether a protein sequence contains a particular PROSITE motif or not. Furthermore, the motif-based compatibility between a query sequence and TIM-barrel fold can be expressed as:

where *S(TIM|motif) *was calculated from equation 2 and summation was performed over all motifs found in the query sequence and fulfilling the following criteria:

where *C *is an adjustable parameter, with 0.1 being a preliminary optimized value in this work. For a given protein sequence, a larger value of *S*_*motif*_(*TIM*|*sequence*) means a higher chance that the sequence is a TIM-barrel protein. Therefore, *S*_*motif*_(*TIM*|*sequence*) is used as the motif-based descriptor.

### Evaluation of individual descriptors

Based on the SCOP_10_mod dataset, the three descriptors' performance in recognizing TIM-barrel proteins was individually assessed. To assess the performance of the PSI-BLAST-based descriptor, a Leave-One-Out analysis was carried out. Each time, a TIM-barrel protein was selected as a "test" protein. By calculating the similarity scores (*i.e. evalue_mod(A, B)*), the "test" protein was searched against all other TIM-barrel proteins in the SCOP_10_mod dataset and the protein with the most significant similarity score (i.e., the top hit) was recorded. Likewise, the non-TIM-barrel proteins were also searched against all TIM-barrel proteins. The top hits and the corresponding *evalue_mod(A, B) *scores were also recorded. By defining a threshold value, the TIM-barrel identification accuracy was measured by Sensitivity and Specificity with definitions as below.

Moreover, a ROC curve, which plots TPR (i.e., Sensitivity) as a function of FPR (*i.e*., 1-Speficity) for all possible thresholds, was also employed to measure the performance. The AUC was also calculated to provide a comprehensive understanding of the performance of the PSI-BLAST-based descriptor. Generally, the closer the AUC value is to 1, the better the descriptor is. The SSEA-based descriptor (i.e., the *SSEA(A, B) score) *was evaluated based on the same strategy.

Regarding the motif-based descriptor, the score *S*_*motif*_(*TIM*|*sequence*) for each protein within the SCOP_10_mod dataset was calculated. Because *S*_*motif*_(*TIM*|*sequence*) reflects a given sequence's compatibility with the TIM-barrel fold, it was directly used to judge whether a given protein should have the TIM-barrel fold.

### Construction of TIM-Finder

#### SVM learning

In this work, the three descriptors were combined into a prediction system called TIM-Finder with the assistance of the SVM algorithm. As a machine-learning method for two classes of classification, SVM aims to find a rule that best maps each member of a training set to the correct classification [41,42]. Here, the SVM was trained to distinguish two different protein pairs related to TIM-barrel proteins. In the first type of protein pair (i.e., positive sample), both proteins are TIM-barrel proteins. The SCOP_10_mod dataset contains 13,203 positive samples [i.e., (163 × 162)/2 = 13,203 pairs; N.B. the pair (*A*, *B*) is the same as (*B*, *A*) in this case]. In the second type of protein pair (i.e., negative sample), the first protein is of TIM-barrel fold but the second one belongs to a non-TIM-barrel protein. Thus, the SCOP_10_mod contains 137,409 negative samples [i.e., 843 × 163 = 137,409 pairs; N.B. the pair (*A*, *B*) is not the same as (*B*, *A*) in this case].

Due to the direction in which the PSI-BLAST search is carried out, the search for *A *against *B *is different from the search for *B *against *A*. In our work, the PSI-BLAST search for sequence *B *against *A *was also carried out. Thus, four parameters (i.e., *evalue_mod(A, B)*, *Score(A, B)*, *evalue_mod(B, A) *and *Score(B, A)*) were generated from the PSI-BLAST-based descriptor. The SSEA descriptor provides one parameter (i.e., *SSEA(A, B)*). Regarding the motif-based descriptor, two parameters (i.e., *S*_*motif*_(*TIM*|*sequence A*) and *S*_*motif*_(*TIM*|*sequence B*) were used. Thus, a total of seven parameters were used in the SVM learning.

The SCOP_10_mod dataset can be compiled into 150,612 protein pairs, which were further divided into 5 roughly equal subsets. An evaluation similar to 5-fold cross-validation was performed. To predict whether a given protein pair belongs to the first type or the second type, the subset to which this pair belongs was labeled as the "test" set, whereas the four remaining subsets were labeled as "training" sets. SVM models were developed for each of the "training" sets. The class label for positive (i.e., the first type) and negative (i.e., the second type) samples was set to +1 and -1, respectively. The ratio of positive to negative samples was 1:10 in the training set. Using the training set at such a ratio would inevitably cause the SVM model to predict every pair as a negative case. The optimized ratio in the training set was set at 1:2.5. Each training set was modified by discarding a random selection of the negative samples prior to training. The training resulted in four separate SVM models, with the predicted score being obtained as an average value over the scores from the four different SVM models.

The implemented SVM algorithm was LIB-SVM http://www.csie.ntu.edu.tw/~cjlin/. The applied kernel function was the radial basis function (RBF). The corresponding parameter settings of SVM learning were automatically optimized by LIB-SVM.

It is worth mentioning here that the predicted score for each protein pair can be regarded as a combination of the corresponding seven parameters with the assistance of SVM. Based on the predicted scores, the performance of TIM-Finder was assessed in the same way as we evaluated the individual descriptors.

#### Web server of TIM-Finder

To facilitate the community's research, a web server of TIM-Finder was constructed and is freely available at http://202.112.170.199/TIM-Finder/. To sufficiently represent the known structural TIM-barrel proteins as well as allow a reasonable computational time, the 322 TIM-barrel proteins in the SCOP_40 dataset were used as the library in the TIM-Finder system. To search a query sequence against the TIM-barrel library (i.e., SCOP_40_TIM), a total of 322 protein pairs are involved. For each protein pair, the corresponding seven parameters are calculated. Then, the resulting seven parameters are used as the input for the five SVM models trained in the above section, and the predicted score is obtained as an average value over the scores from the five different SVM models. Generally, the predicted score reflects the query sequence's probability of adopting a TIM-barrel fold. Finally, the predicted scores for all protein pairs are ranked, and the top 10 hits are reported. In the resulting page provided by TIM-Finder, the SCOP entry number, PDB link, prediction score, and the corresponding confidence level for each of the top 10 hits are listed. The whole process for each query normally takes about 10 minutes with a single processor on our Red Hat Enterprise Linux 5 system.

To provide confidence levels for different prediction scores resulting from TIM-finder, a stringent negative dataset based on the SCOP_40 dataset was compiled. First, in the initial SCOP_40 dataset only the non-TIM-barrel proteins that belong to α/β class (i.e., the same structural class as the TIM-barrel fold) were kept. Second, the proteins with a sequence length < 100 or > 1000 were removed. Third, the proteins that had been used in training TIM-Finder (i.e., the five SVM models) were further discarded. Finally, 1,999 non-TIM-barrel proteins retained. We processed all 1,999 proteins on TIM-Finder, and it was estimated that a prediction score = 0.82 yields a ≤ 1% FPR (i.e., 99% confidence level) and a prediction score = 0.38 indicates a ≤ 5% FPR (i.e., 95% confidence level). Compared with proteins from other structural classes, query proteins belonging to the α/β class should have a higher probability of being predicted as TIM-barrel proteins. We only selected the α/β proteins as negative controls, which should guarantee a reliable estimate of thresholds for different confidence levels.

### Construction of the amino acid composition based SVM model

The AAC-based SVM model (i.e., AAC_SVM) was trained to distinguish TIM-barrel and non-TIM-barrel proteins. Briefly, the 163 TIM-barrel proteins in the SCOP_10_mod dataset were considered positive instances and their labels were set to + 1, while 843 non-TIM-barrel proteins were considered negative instances and their labels were set to - 1. The AAC for each protein was used as the input feature vector. A 10-fold cross-validation was performed. We divided SCOP_10_mod into 10 roughly equal subsets. In each evaluation step, one subset was selected for testing, while the rest nine subsets were merged into a training dataset. LIB-SVM with the RBF kernel was employed to train the SVM models, and the other SVM parameter settings were also automatically optimized by LIB-SVM. Based on the predicted SVM scores, AAC_SVM was assessed in the same way as TIM-Finder.

### Proteome-wide TIM-barrel protein identification based on TIM-Finder, Fugue and PSI-BLAST

To benchmark the performance of TIM-Finder, Fugue, and PSI-BLAST, the proteome-wide TIM-barrel protein identification in *B. subtilis *was carried out. The whole proteome of *B. subtilis*, which contains 4,102 protein sequences, was obtained from ftp://ftp.ncbi.nlm.nih.gov/genomes/Bacteria. The *B. subtilis *proteins with a sequence length <100 or >l000 amino acids were ruled out in our analysis, because they have less chance to be TIM-barrel proteins or a high possibility of containing more than one domain. Thus, 3,575 sequences were kept for further analysis.

TIM-Finder was performed on these 3,575 sequences via the established TIM-Finder server. The stand-alone version of Fugue [13] was provided by Dr. Kenji Mizuguchi (National Institute of Biomedical Innovation, Japan), and the corresponding fold library (i.e., the HOMSTRAD database) in its version of 05/2008 was downloaded from http://tardis.nibio.go.jp/homstrad/, which consists of 4,026 representative protein structures. The 3,575 protein sequences were processed by Fugue, and the top hits as well as the corresponding Z-scores were generated for each query sequence. As suggested by Fugue developers, a Z-score = 6.0 corresponds to a 99% confidence level and a Z-score = 4.0 indicates a 95% confidence level. For comparison, the PSI-BLAST search was also performed on these 3,575 protein sequences. As in deriving the PSI-BLAST-based descriptor, each sequence was first searched against the NR90 database by PSI-BLAST for three rounds to generate a profile. Then a PSI-BLAST search was performed on the obtained profile against the SCOP_40_TIM sequences for another round and the top hit was recorded. Based on the same procedure as we used to define the confidence levels of TIM-Finder prediction scores, it was estimated that an *e*-value ≤ 0.009 means a 99% confidence level and an *e*-value ≤ 0.066 indicates a 95% confidence level.

## Availability and requirements

**Project Name**: TIM-Finder

**Project home page**: http://202.112.170.199/TIM-Finder/

**Operating system**: Online service is web based; local version of the software should be run in a Linux platform.

**Programming language**: Perl.

**Other requirements**: None.

**License**: Free.

**Any restrictions to use by non-academics**: None.

## Authors' contributions

JN wrote programs, constructed the website and drafted the manuscript. RX and CW participated in the analysis of data and the construction of website. ZZ and XDS conceived of the study. ZZ directed the research and critically revised the manuscript. All the authors have read and approved the final manuscript.
